# Supplementary material for: Chromosomal instability induced in cancer can enhance macrophage-initiated immune responses that include anti-tumor IgG
Source: eLife. 2024 May 28;12:RP88054. doi: 10.7554/eLife.88054 (PMC11132682; doi:10.7554/eLife.88054)
Supplement: MDAR checklist [file elife-88054-mdarchecklist1.pdf]

Hayes...Discher
eLife-RP-RA-2023-88054R1

Materials Design Analysis Reporting (MDAR) Checklist for Authors

The MDAR framework establishes a minimum set of requirements in transparent reporting mainly applicable to studies in the life sciences.

eLife asks authors to **provide detailed information within their article** to facilitate the interpretation and replication of their work. Authors can also upload supporting materials to comply with relevant reporting guidelines for health-related research (see EQUATOR Network), life science research (see the BioSharing Information Resource), or animal research (see the ARRIVE Guidelines and the STRANGE Framework; for details, see *eLife*'s Journal Policies). Where applicable, authors should refer to any relevant reporting standards materials in this form.

For all that apply, please note **where in the article** the information is provided. Please note that we also collect information about data availability and ethics in the submission form.

Materials:

Newly created materials	Indicate where provided: section/figure legend	N/A
There are no unique materials created.		N/A

Antibodies	Indicate where provided: section/figure legend	N/A
See Table in Materials & Methods	Methods section	

DNA and RNA sequences	Indicate where provided: section/figure legend	N/A
Seqeunces are included.	Methods section	

Cell materials	Indicate where provided: section/figure legend	N/A
Seqeunces are included.	Methods section	
Primary cultures.		N/A

Experimental animals	Indicate where provided: section/figure legend	N/A
Seqeunces are included.	Methods section	
Animal observed in or captured from the field		N/A

Plants and microbes	Indicate where provided: section/figure legend	N/A
		N/A
		N/A

**Human research participants**	**Indicate where provided: section/figure legend) or state if these demographics were not collected**	**N/A**
		N/A

Design:

**Study protocol**	**Indicate where provided: section/figure legend**	**N/A**
		N/A

**Laboratory protocol**	**Indicate where provided: section/figure legend**	**N/A**
		N/A

**Experimental study design (statistics details) ***		
**For in vivo studies: State whether and how the following have been done**	**Indicate where provided: section/figure legend. If it could have been done, but was not, write “not done”**	**N/A**
Sample size determination		
Randomisation		
Blinding		
Inclusion/exclusion criteria		

**Sample definition and in-laboratory replication**	**Indicate where provided: section/figure legend**	**N/A**
State number of times the experiment was replicated in the laboratory.	Experiments were typically replicated at least 3 times. Sample sizes are shown in Tables and Figure legends, and represent biological replicates (individual cells or animals).	

Data describe technical or biological replicates.	Data describe individual animals as biological replicates.	

Ethics	Indicate where provided: section/submission form	N/A
		N/A
Studies involving experimental animals: State details of authority granting ethics approval (IRB or equivalent committee(s), provide reference number for approval.	Research involving vertebrate animals was done at the University of Pennsylvania following protocols reviewed and approved by the Institutional Animal Care and Use Committee (IACUC). The animals were cared for by Veterinary Services under a currently AAALAC approved program. The animals were housed in NIH-approved facilities and are observed daily by technicians. Unusual events are reported to the on call veterinarian, as well as to the investigator according to posted protocols. Other maintenance veterinary care was conducted according to NIH guidelines on the Use and Care of Animals. Facilities were inspected regularly according to NIH and AAALAC guidelines.	
Studies involving specimen and field samples: State if relevant permits obtained, provide details of authority approving study; if none were required, explain why.		N/A

Dual Use Research of Concern (DURC)	Indicate where provided: section/submission form	N/A
If study is subject to dual use research of concern regulations, state the authority granting approval and reference number for the regulatory approval.		N/A

Analysis:

**Attrition**	**Indicate where provided: section/figure legend**	**N/A**
Describe whether exclusion criteria were pre-established. Report if sample or data points were omitted from analysis. If yes, report if this was due to attrition or intentional exclusion and provide justification.	All data generated or analyzed are included in the figures.	

**Statistics**	**Indicate where provided: section/figure legend**	**N/A**
Describe statistical tests used and justify choice of tests.	See the Methods section entitled “Statistical analysis”.	

**Data availability**	**Indicate where provided: section/submission form**	**N/A**
For newly created and reused datasets, the manuscript includes a data availability statement that provides details for access (or notes restrictions on access).	All data associated with this study are present in the paper or the Supplementary Materials.	
When newly created datasets are publicly available, provide accession number in repository OR DOI and licensing details where available.		N/A

**Code availability**	**Indicate where provided: section/figure legend**	**N/A**
		N/A

		N/A

Reporting:

The MDAR framework recommends adoption of discipline-specific guidelines, established and endorsed through community initiatives.

**Adherence to community standards**	**Indicate where provided: section/figure legend**	**N/A**
State if relevant guidelines (e.g., ICMJE, MIBBI, ARRIVE, STRANGE) have been followed, and whether a checklist (e.g., CONSORT, PRISMA, ARRIVE) is provided with the manuscript.		N/A
